# Hsa_circRNA_102002 facilitates metastasis of papillary thyroid cancer through regulating miR-488-3p/HAS2 axis

**DOI:** 10.1038/s41417-020-00218-z

**Published:** 2020-08-29

**Authors:** Wei Zhang, Ting Liu, Tianshu Li, Xudong Zhao

**Affiliations:** 1 Department of Endocrinology, Shengjing Hospital of China Medical University, 110000 Shenyang City, Liaoning Province China; 2 Department of Otolaryngology Head and Neck Surgery, Shengjing Hospital of China Medical University, 110000 Shenyang City, Liaoning Province China

**Keywords:** Biotechnology, Cancer

## Abstract

As important modulators in various physiological processes, circular RNAs (circRNAs) have been increasingly demonstrated in tumors, including papillary thyroid cancer (PTC). Hsa_circRNA_102002 (circ_102002) is a circRNA derived from alternative splicing of ubiquitin-specific peptidase 22 (USP22) transcript, the role of which needs further investigation. Our results suggested the upregulation of circ_102002 in PTC tissues and cells, and its promoting effects on epithelial–mesenchymal transition (EMT) and cell migration. Mechanism studies showed that circ_102002 could sponge microRNA-488-3p (miR-488-3p) and downregulate its expression. The target relationship between miR-488-3p and hyaluronic acid synthetase 2 (HAS2) in PTC was systematically studied. In addition, our results showed that HAS2 overexpression could restore the inhibited cell EMT and migration. Moreover, the inhibitory effect of downregulation of circ_102002 on PTC growth was evaluated in a mouse xenograft model, which involved miR-488-3p and HAS2 regulation. These findings about the signal axis of circ_102002/miR-488-3p/HAS2 may further elucidate the PTC pathogenesis and improve clinical treatment.

## Introduction

Papillary thyroid cancer (PTC) is the most common endocrine malignant tumor, representing ~90% of all thyroid cancers [1]. Although the detection and treatment methods have made some progress and improved the prognosis, metastases commonly involve cervical lymph nodes and lung [2]. Lung metastasis occurs in 7–30% in children and adolescents (≤20 years), only ~4% in adult population [3, 4]. These PTC patients still suffered from poor outcomes. Therefore, exploration of the molecular mechanism related to the pathogenesis of PTC metastasis may offer alternative intervention targets and improve the therapeutic strategies.

CircRNAs have been identified as endogenous noncoding RNAs, which are widely found in eukaryotes and possess tissue specificity [5–7]. Characterized as a covalently closed continuous loop, circRNA is resistant to cleavage of RNA exoribonuclease, making it a regulator of microRNA (miRNA) binding, protein interaction and regulatory splicing [8, 9]. The characteristics and functions of circRNAs are increasingly demonstrated in PTC. For example, circ_0067934 was highly expressed in PTC tissues, while circ_0067934 knockdown inhibited epithelial-mesenchymal transition (EMT) and PI3K/AKT signaling pathways [10]. In addition, circRNA_102171 promotes PTC progression by modulating the activation of CTNNBIP1-dependent β-catenin pathway [11]. Hsa_circRNA_102002, derived from alternative splicing of ubiquitin-specific peptidase 22 (USP22) transcript, is profiled to be upregulated in PTC tissues by microarray [12]. Although USP22 has been reported to be related to glioma progression [13], colorectal cancer drug resistance [14], and PTC metastasis [15], the role of hsa_circRNA_102002 in PTC has not been fully recognized. Serving as epigenetic regulators, circRNAs usually functions by sponging miRNAs, which further regulate the expression of complementary messenger RNAs and impart cancer development. Recent studies indicated that miR-488-3p functioned as a tumor suppressor in esophageal squamous cell carcinoma by targeting zinc finger and BTB domain containing 2 (ZBTB2) and activating p53 pathway [16]. Moreover, miR-488-3p could regulate protein kinase, DNA-activated, catalytic subunit (PRKDC) and sensitize malignant melanoma cells to cisplatin [17]. However, the function of miR-488-3p and potential target genes in PTC are still unknown.

As a member of hyaluronic acid synthetase (HAS), HAS2 is a transmembrane glycosyltransferase and a major ECM proteoglycan, which plays an important role in the high expression of macromolecular hyaluronic acid during the malignant transformation [18]. In the tumor microenvironment, macromolecular hyaluronic acid provides a hydrated matrix to create gaps in the extracellular matrix that facilitate tumor cell migration [19]. Recent studies have found that hyaluronic acid is involved in a number of cancer processes, including multidrug resistance and angiogenesis. HAS2 was found to promote ZEB1-mediated EMT and metastasis in breast cancer [19]. In addition, HAS2 could activate the PI3K/Akt pathway and increase the invasion capacity of breast cancer cells by inhibiting timp-1 and promoting FAK phosphorylation [20]. Moreover, the function of HAS2 in promoting tumor progression have been demonstrated in pancreatic cancer [21], colorectal cancer [22] etc. However, the regulatory effects and mechanisms of HAS2 in PTC remains to be elucidated.

In this study, we determined the circ_102002 expression in PTC tissues and explored the effect of circ_102002 on EMT and cell migration. Potential target miRNAs by circ_102002 were predicted by starBase and Circular RNA Interactome. Based on target prediction analysis, we found that miR-488-3p might target the 3ʹ-UTR of HAS2. Therefore, it can be speculated that circ_102002 might promote PTC EMT and metastasis by sponging miR-488-3p and upregulating HAS2 expression. The regulatory manner of circ_102002/miR-488-3p/HAS2 axis in PTC was systematically studied, which may help to develop new candidates for PTC diagnosis and therapy.

## Materials and methods

### Patient tissue samples

Paired papillary thyroid cancer (PTC) tissues (tumor) and normal adjacent tissues (normal) samples were collected at Shengjing Hospital of China Medical University. No chemotherapy or radiotherapy was performed for these PTC patients before section. The experiments were under the permission of the Ethics Committee of Shengjing Hospital of China Medical University (Approval No. 2019PS100J). Written informed consents were obtained from patients. Three pathologists performed diagnostics independently. Patient clinical information was listed in Table 1.

**Table 1:** Relationship between has_circ_102002 expression and the clinical pathological characteristics of PTC patients.

Clinical pathological features	No. of cases	Hsa_circ_102002 expression		*p-*value
		Low expression (< median)	High expression (≥ median)	
Number	50	25	25	
Gender				>0.05
Male	29	15	14	
Female	21	10	11	
Age (years)				>0.05
≤45	36	16	20	
>45	14	9	5	
Tumor size (cm)				>0.05
≤1	37	21	16	
>1	13	4	9	
Lymph node metastasis				<0.05
≤45	23	17	6	
>45	27	8	19	
Nodular goiter				>0.05
Negative	33	15	18	
Positive	17	10	7	
TNM stage				<0.05
I/II	29	21	8	
III/IV	21	4	17	
Extra thyroidal extension				>0.05
Negative	15	5	10	
Positive	35	20	15	

### RNA extraction, microarray analysis, and quantitative real-time PCR (qRT-PCR)

Total RNAs were extracted from PTC cells and tissues using TRIzol reagent (Thermo Fisher Scientific, Rockford, IL, USA). The RNA quality was evaluated by electrophoresis in agarose and observed by Agilent 2100 Bioanalyzer (Agilent Technologies, Chandler, USA). Microarray of circRNAs was performed in BGI.Tech (Beijing, China) and analyzed according to the Arraystar. Nucleus and cytoplasm were separated using an extraction Kit (Inventbiotech, Beijing, China). Complementary DNA was synthesized from total RNA by using PrimeScript RT Reagent Kit (Takara, Shiga, Japan). qRT-PCR assays for genes were conducted by SYBR Premix Ex Taq (Takara). SYBR PrimeScript miRNA RT-PCR Kit (Takara) was applied to quantity miRNA expressions. GAPDH (glyceraldehyde-3-phosphate dehydrogenase) is an enzyme in the classic glycolysis reaction, accounting for 10–20% of total proteins. The GAPDH gene has a highly conserved sequence and its expression in the same cell or tissue is generally constant. To normalize circ_102002 and HAS2 mRNA levels, GAPDH was selected as endogenous control. U6 snRNA was used to normalize miRNA expressions. After the Ct values of GAPDH or U6 in each sample were obtained by qRT-PCR, the stability of the reference genes was analyzed by internal reference screening software (geNorm, NormFinder and Bestkeeper). The amount of RNA to relative to the reference gene was calculated using the equation 2^–ΔΔCT^. The corresponding primer sequences were listed in Table S1.

### Overall survival analysis

After qRT-PCR analysis, the average circ_102002 level was calculated and used as a criterion to evaluate the expression of circ_102002. If circ_102002 level exceeds the average level, high expression level was identified. When the circ_102002 level was under the average value, low expression level was classified. According to patients’ follow-up data, overall survival curves were analyzed by the Kaplan–Meier method using log-rank test.

### In-situ hybridization (ISH)

An ISH kit (Thermo Fisher Scientific) was used to perform fluorescence detection of circ_102002 according to the manufacturer’s instructions. Tissues were first fixed in paraformaldehyde and then embedded using paraffin. After that, sections (5 μm) were cut. For tissue ISH, circ_102002 probe was labeled with peroxidase (Thermo Fisher Scientific). For cell ISH, cells were fixed in paraformaldehyde, blocked in pre-hybridization buffer, incubated with a circ_102002 probe conjugated by FITC (Exiqon, Vedbaek, Denmark) in hybridization buffer, and finally stained by DAPI (Sigma-Aldrich). Fluorescence images were photographed by a confocal microscope (Nikon, Tokyo, Japan). During the experiments of ISH, tissue sections hybridizated with mismatch control probe instead of circ_102002 probe were used as negative controls.

### Immunofluorescence (IF) and immunohistochemistry (IHC) assays

Tissue sections were first cut. Since methylene bridge was generated in the process of formaldehyde fixation, the proteins were cross-linked to screen the antigen sites. The sections were immersed in citrate repair solution, and then heated in the microwave until boiling. The subboiling temperature (95–98 °C) was maintained for 10 min, and the antigens were reexposed by heat to restore the original conformation of the protein for immunohistochemical staining. For IF assay, antibodies against COX-2 and Ki67 were used to incubate with tissue sections overnight at 4 °C. Then, sections were incubated with secondary antibodies conjugated by Cy5.5 or FITC (Exiqon, Vedbaek, Denmark) in hybridization buffer, and finally stained by DAPI (Sigma-Aldrich). Fluorescence images were photographed by a confocal microscope (Nikon). For IHC assay, corresponding secondary antibodies and 3, 3ʹ-diaminobenzidine (DAB) solution (Sigma-Aldrich, St. Louis, MO, USA) were used to visualize the sections. Moreover, a microscope (TE2000-U, Nikon, Japan) was used to capture the IHC images. Tissue sections stained with isotype matched IgG instead of HAS2 primary antibody were used as negative controls.

### Cell culture

Human normal thyroid cells (HTori-3) are immortalized by transfecting primary cultures of human thyroid epithelial cells with an origin-defective SV40 genome [23]. KTC-1 and CAL62 cells were purchased from Shanghai Institutes for Biological Sciences, Chinese Academy of Sciences (Shanghai, China). TPC-1 cells were obtained from Procell Life Science&Technology (Wuhan, China). SNU-790 cells were purchased from Korean Cell Line Bank (KCLB, Seoul, Korea). All cells were identified by short tandem repeat (STR) genotyping. For HTori-3 cells culture, F-12K Medium was used. Moreover, RPMI-1640 Medium was used for TPC-1, KTC-1, CAL62, and SNU-790 cells. All the culture media were supplemented with 10% FBS (Thermo Fisher Scientific), and all the cells were cultured in a humidified atmosphere with 5% CO_2_ at 37 °C. The cellular morphology of SNU-790 cells with circ_102002 upregulation or TPC-1 with circ_102002 downregulation was captured by a microscope (Nikon).

### Cell infection and cell transfection

Lentivirus expressing circ_102002 (Lv-circ_102002), expressing shRNA for circ_102002 (sh- circ_102002) and their corresponding controls (Lv-NC or sh-NC) were purchased from GenePharma (Shanghai, China) and infected SNU-790 or TPC-1 cells. Puromycin (300 µg/ml, Sigma-Aldrich) was used for screening stable cells infected with these lentivirus, and single clones were harvested. In addition, cell transfection was performed with Lipofectamine 2000 (Invitrogen) according to the manufacturer’s instructions. SNU-790 or TPC-1 cells were transfected with miR-488-3p mimics (miR-488-3p), miR-625-5p mimics (miR-625-5p), miR-1197 mimics (miR-1197), miR-488-3p inhibitors (miR-488-3p inh), HAS2 expressing plasmid (HAS2) or the relevant controls (NC mimics, NC inh or Vector). The protocol for cell transfection in a 6-well plate is as follows: plate 5 × 10^4^ cells per well in 2 ml of complete growth medium 12 h prior to transfection; wash with 1 × PBS and add 2 ml of fresh Opti-MEM medium; prepare transfection complexes by mixing 40 µl of serum-free medium, 6 µl of Lipofectamine 2000, and 1000 ng DNA or 100 nM miRNA mimics (or inhibitors); incubate transfection complexes at room temperature for 20 min; add prepared transfection complexes to 2 ml of Opti-MEM medium; incubate cells at 37 °C in a humidified CO_2_ incubator; assays for phenotype or target gene expression were performed 48–72 h after transfection. miRNA mimics and inhibitors were chemically synthesized (GenePharma). HAS2 expressing plasmid was constructed by enzyme digestion and ligation, and was confirmed by DNA sequencing (GenePharma).

### Western blotting

Protein was extracted from PTC cells by RIPA lysis buffer (Sigma-Aldrich). Protein quantification was performed by a BCA protein assay kit (Thermo Fisher Scientific). After that, 50 µg total proteins from each sample) were separated by sodium dodecyl sulfate-polyacrylamide gel electrophoresis (SDS-PAGE), and transferred onto polyvinylidene fluoride membranes (Millipore, Bedford, MA, USA), Then, immunoblot analyses was conducted by incubation with primary antibodies (overnight at 4 °C), including Vimentin, Slug, Twist, MMP2, MMP9, HAS2, p-FAK, FAK, p-AKT, and AKT. After that, membranes were incubated with a horseradish peroxidase-conjugated secondary antibodyand and visualized using the enhanced chemiluminescence kit (Santa Cruz, Dallas, TX, USA). GAPDH was used as internal control and protein bands were quantified using Quantity One software (Bio-Rad, Berkeley, CA, USA). For antibody information, see Supplementary Table S2.

### Wound scratch assay

Linear scratch wounds were conducted on the cell monolayer. After 24 h, an inverted microscope (Nikon) was subjected to take photos of cell migration. Then, the distances of the scrape were determined using an Image J software (http://rsb.info.nih.gov/ij/).

### Transwell assay

For cell migration assay, SNU-790 or TPC-1 cells (1 × 10^5^) were cultured in the top chamber of an insert (Corning Costar Co., Cambridge, MA, USA). For cell invasion assay, the insert was pre-coated by Matrigel (working concentration: 2 mg/ml, BD Biosciences, San Jose, CA). In the top chamber, cell culture was performed using serum-free medium. Cell culture medium with FBS (10%) was applied to induce cells migrating to the lower chamber of the insert. Twelve hours later, the migrated and invaded cells were fixed. The matrigel and cells in the upper chamber were swabbed away by a cotton swab. Moreover, cells underside of the membrane were then stained with 0.1% crystal violet, and imaged by a microscope (Nikon). The number of cells in ten different fields was counted and compared with the control group.

### RNA binding protein immunoprecipitation (RIP) assay

RIP assay was performed as previously reported [24]. Ago2 antibodies or IgG were used to incubate the cell lysates of SNU-790 or TPC-1 cells. RNA was extracted from the pull down complexes, and relative levels of miR-488-3p, miR-625-5p, and miR-1197 were then determined using qRT-PCR.

### Target prediction analysis

The potential target genes of miR-488-3p were analyzed using four miRNA prediction software, including Targetscan (http://www.targetscan.org/vert_71), mirTarBase (http://mirtarbase.mbc.nctu.edu.tw/php/index.php), miRDB (http://www.mirdb.org/), and starBase (http://starbase.sysu.edu.cn). Ten genes (HAS2, LRRC4A, BRPF1, EIF3A, VPS4B, ZNF281, ARPP19, PKNOX1, ANAPC16, and STK4) were predicted by the intersection analysis, and were studied as the potential target genes of miR-488-3p.

### Luciferase reporter assay

Luciferase reporter assays was conducted as previously reported [25]. When cell confluence reached 70%, β-galactosidase expression vector (Promega), firefly luciferase reporter plasmid and miR-488-3p inh, NC inh, miR-488-3p mimics or NC mimics were co-transfected into SNU-790 or TPC-1 cells. By means of a Luciferase Reporter Assay System (Promega), luciferase activities were determined after 24 h.

### Plasmid construction

The sequence of circ_102002 was amplified using a human genomic DNA and inserted into the p-MIR-reporter plasmid (Promega, Madison, WI, USA). The mutant luciferase plasmid (binding site: UGGCCUUUCA substituted by AUCGGAAAGU) was also used as a control. According to the methods above, the p-MIR-reporter plasmid containing the 3ʹ-UTR of HAS2 was obtained. In addition, the corresponding mutant luciferase plasmid (binding site: UCAAGGAAAAGUUCUUUCA substituted by UCAAGAAAAGUUGAAAGU) was constructed as a negative control. Primers sequences were listed in Table S3.

### Mouse lung metastasis model

Animal experiments were operated according to the Guidelines for the Care and Use of Laboratory Animals published by the National Institutes of Health [26] and approved by the Ethics Committee of Shengjing Hospital of China Medical University. To establish a PTC lung metastasis model, thymic BALB/c nude mice (16–18 g) were used. Stable TPC-1 cells expressing a luciferase and shRNA for circ_102002 (sh-circ_102002) or the control cells (sh-NC) were used. Mice (5 mice/group) were intravenously injected with these cells (1 × 10^6^). At 21 days post-implantation, the tumor-bearing mice were intraperitoneally injected with 100 μl of d-luciferin (10 mg/ml in PBS, Promega, Madison, WI, USA). After 10 min, the fluorescence images of the tumor and lung metastasis in the mice were imaged under anesthesia with 2.5% isofluorane using an IVIS Lumina XR III in vivo imaging system (PerkinElmer, Waltham, CA). Luminescence was expressed as photons/s per region of interest minus background luminescence for a similarly sized region. The lungs were dissected from mice and photographed. Additionally, haematoxylin and eosin (H&E) staining and IHC analysis of HAS2, p-FAK, p-Akt, E-cadherin, and N-cadherin in the tumor tissues were, respectively, conducted. All the antibody information was listed in Supplementary Table S2.

### Statistical analysis

Experiments were performed in triplicate and repeated for at least three times independently. Samples and animals were randomly allocated to experimental groups and processed, and was blind with the investigator. Data were checked and they obeyed a normal distribution. Results were presented as the mean ± standard error of the mean (SEM). Student’s *t*-test was used to analyze the statistical differences between two groups, and one-way analysis of variance was used for multiple groups. The correlations between circ_102002 and miR-488-3p, circ_102002 and HAS2 mRNA, miR-488-3p and HAS2 mRNA were, respectively, determined by Pearson correlation analysis. *p* < 0.01 was classified as significant. */# *p* < 0.05; **/# # *p* < 0.01.

## Results

### Hsa_circ_102002 was upregulated in papillary thyroid carcinoma tissues and cells

**Fig. 1:**
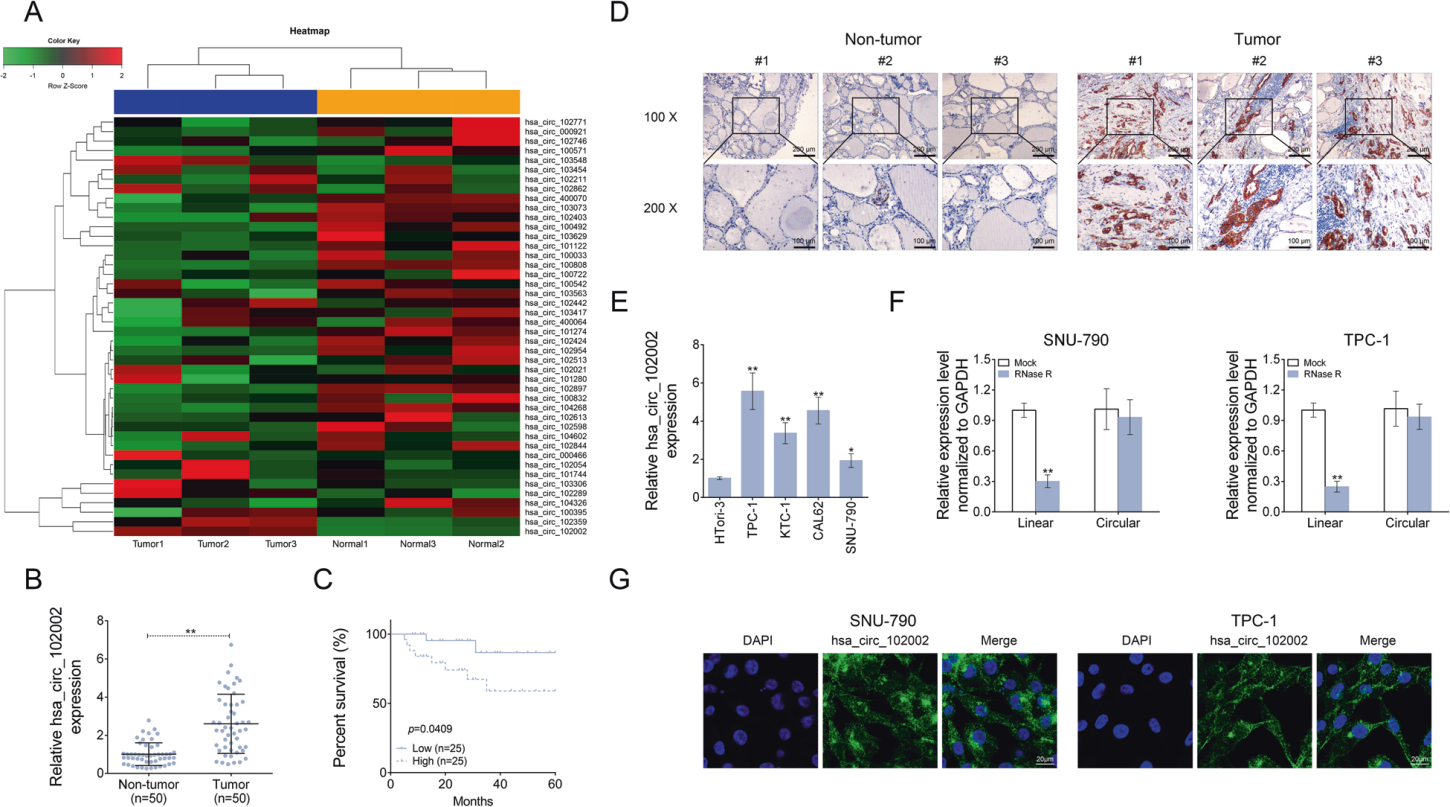
Hsa_circ_102002 was upregulated in papillary thyroid carcinoma tissues and cells. **a** The heatmap of differently expressed circle RNAs in PTC tissues (tumor, *n* = 3) and normal adjacent tissues (non-tumor, *n* = 3). **b** Relative hsa_circ_102002 expression levels in PTC tissues (tumor, *n* = 50) and normal adjacent tissues (non-tumor, *n* = 50), as determined using qRT-PCR. **c** Kaplan–Meier curves of overall survival of 50 PTC patients, stratified by hsa_circ_102002 expression. **d** Representative images of PTC tissues (tumor, *n* = 50) and normal adjacent tissues, as determined using ISH. Original magnification, ×40 and ×400. **e** Relative hsa_circ_102002 expression levels in human normal thyroid cells (HTori-3) and four thyroid cancer cell lines (TPC-1, KTC-1, CAL62, and SNU-790), as determined by qRT-PCR. **f** Relative linear RNA and circular RNA expression levels in SNU-790 cells after RNase R treatment, as determined using qRT-PCR. **g** Subcellular localization of hsa_circ_102002 in SNU-790 or TPC-1 cells, as determined using ISH. (mean ± SEM, *p < 0.05, **p < 0.01).

To explore the role of circRNAs in papillary thyroid cancer, circRNA microarray was used to determine differently expressed circRNAs in PTC tissues (tumor, *n* = 3) and normal adjacent tissues (non-tumor, *n* = 3). Among them, hsa_circ_102002 was found to be the most significant differentially expressed circRNA in PTC samples compared to normal samples (Fig. 1a). After that, 50 pairs of PTC tissues and normal adjacent tissues were used to analyze hsa_circ_102002 expression by qRT-PCR. The results showed that hsa_circ_102002 was remarkably upregulated in PTC tissues (Fig. 1b). As shown in Fig. 1c, PTC patients with high hsa_circ_102002 expression had a relatively lower survival rate. Patients in high hsa_circ_102002 expression group had a higher probability of lymph node metastasis and distant metastases, and their 5-year overall survival rate was only 50%. While, patients in low hsa_circ_102002 expression group were not prone to metastases, and their 5-year survival rate was close to 90%. In addition, the results of ISH also showed that hsa_circ_102002 was highly expressed in PTC tissues (Fig. 1d). Similarly, the levels of hsa_circ_102002 in PTC cells, such as TPC-1, KTC-1, CAL62 and SNU-790, were higher than that in normal thyroid cells (HTori-3). Since SNU-790 or TPC-1 cells expressed a relatively lower or higher hsa_circ_102002 expression, they were used in the following study (Fig. 1e). RNase R is a ribonuclease and can degrade linear RNA, but is inefficient for circular RNA. As shown in Fig. 1f, RNase R treatment could degrade the linear transcript of USP22, but did not work for hsa_circ_102002. Subcellular localization of hsa_circ_102002 in SNU-790 or TPC-1 cells indicated that hsa_circ_102002 was mainly expressed in cytoplasm (Fig. 1g). Altogether, these results suggested that hsa_circ_102002 is upregulated in PTC tissues and cells, which may exert an important function in PTC progression.

### Hsa_circ_102002 promoted epithelial–mesenchymal transformation of PTC cells

**Fig. 2:**
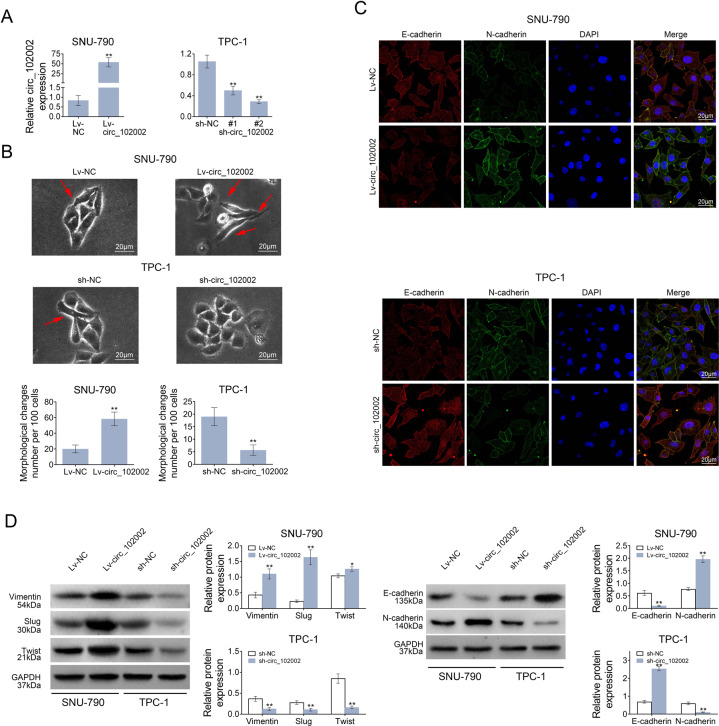
Hsa_circ_102002 could promote epithelial–mesenchymal transformation of PTC cells. **a** Relative hsa_circ_102002 expression levels in Lv-NC or Lv-circ_102002 SNU-790 cells and sh-NC or sh-circ_102002 (sh-circ#1, sh-circ#2) TPC-1 cells. **b** Representative cellular morphology images of Lv-NC or Lv-circ_102002 SNU-790 cells, and sh-NC or sh-circ_102002 TPC-1 cells. The red arrows point to cells with spindle shape. **c** Representative images of E-cadherin and N-cadherin in Lv-NC or Lv-circ_102002 SNU-790 cells and sh-NC or sh-circ_102002 TPC-1 cells, as determined using IF (red, E-cadherin; green, N-cadherin; blue, DAPI). **d** Protein expression levels of E-cadherin, N-cadherin, Vimentin, Slug, and Twist in Lv-NC or Lv- circ_102002 SNU-790 cells and sh-NC or sh-circ_102002 TPC-1 cells, as determined using western blotting. Bands were quantified and shown in histogram. (mean ± SEM, *p < 0.05, **p < 0.01).

To study hsa_circ_102002’s function, SNU-790 cells stably overexpressing hsa_circ_102002 and TPC-1 cells stably downregulating hsa_circ_102002 were constructed and confirmed by q-PCR (Fig. 2a). As shown in Fig. 2b, the cellular morphology of Lv-circ_102002 SNU-790 cells changed to a more obvious spindle shape, and the intercellular mass increased compared to Lv-NC SNU-790 cells. The cellular morphology showed the opposite trend when downregulating hsa_circ_102002 in TPC-1 cells. IF analysis indicated that the expression of E-cadherin was downregulated and the level of N-cadherin was upregulated in Lv-circ_102002 SNU-790 cells. Accordingly, opposite phenomenon was observed in sh-circ_102002 TPC-1 cells (Fig. 2c). In addition, western blot analysis showed that the expression of E-cadherin was reduced and the level of N-cadherin was increased in Lv-circ_102002 SNU-790 cells, while the change trends were reversed in sh-circ_102002 TPC-1 cells. Furthermore, the levels of mesenchymal phenotype biomarkers (Vimentin, Slug and Twist) were increased in Lv-circ_102002 SNU-790 cells, and were reduced in sh-circ_102002 TPC-1 cells (Fig. 2d). All these results suggested that hsa_circ_102002 could promote epithelial–mesenchymal transformation of PTC cells.

### Hsa_circ_102002 could promote cell migration in PTC

**Fig. 3:**
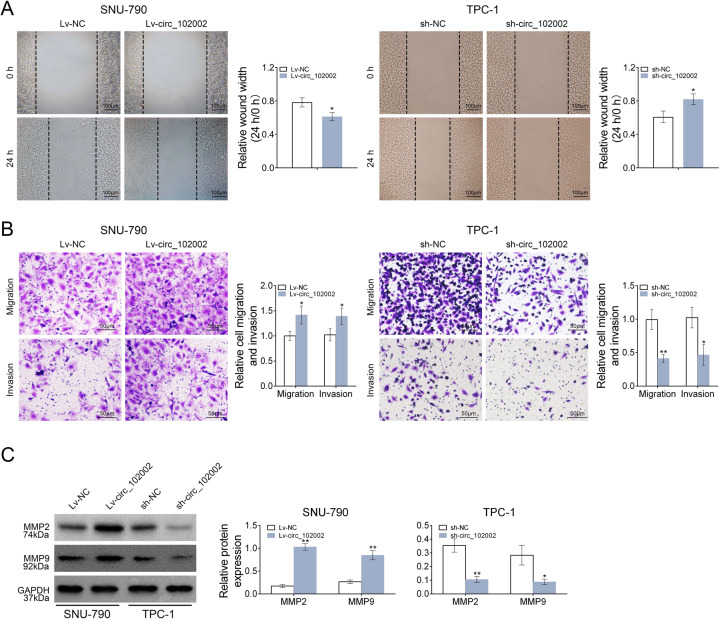
Hsa_circ_102002 could promote the migration and invasion of PTC cells. **a** Wound scratch healing assay of Lv-NC or Lv-circ_102002 SNU-790 cells and sh-NC or sh- circ_102002 TPC-1 cells. Quantification of the wound-healing assay was shown as histograms. **b** Representative migration and invasion assay images of Lv-NC or Lv-circ_102002 SNU-790 cells and sh-NC or sh-circ_102002 TPC-1 cells. The migrated and invaded cells were quantified and shown as histograms. **c** Protein expression levels of MMP2 and MMP9 in Lv-NC or Lv-circ_102002 SNU-790 cells and sh-NC or sh-circ_102002 TPC-1 cells, as determined using western blotting. Bands were quantified and shown in histogram (mean ± SEM, *p < 0.05, **p < 0.01).

The effects of circ_102002 on cell migration were studied by wound scratch healing, transwell and western blot assays. The results indicated that the migration in Lv-circ_102002 SNU-790 cells was promoted, while inhibited in sh- circ_102002 TPC-1 cells (Fig. 3a). Moreover, the number of migrated and invaded Lv-circ_102002 SNU-790 cells was significantly increased, and that of sh- circ_102002 TPC-1 cells was markedly reduced (Fig. 3b). The protein levels of MMP2 and MMP9 were significantly upregulated in Lv-circ_102002 SNU-790 cells, whereas decreased in sh- circ_102002 TPC-1 cells (Fig. 3c). These data above suggested that circ_102002 could promote cell migration in PTC, and its downregulation exerted an inverse effect.

### Circ_102002 sponged miR-488-3p in PTC

**Fig. 4:**
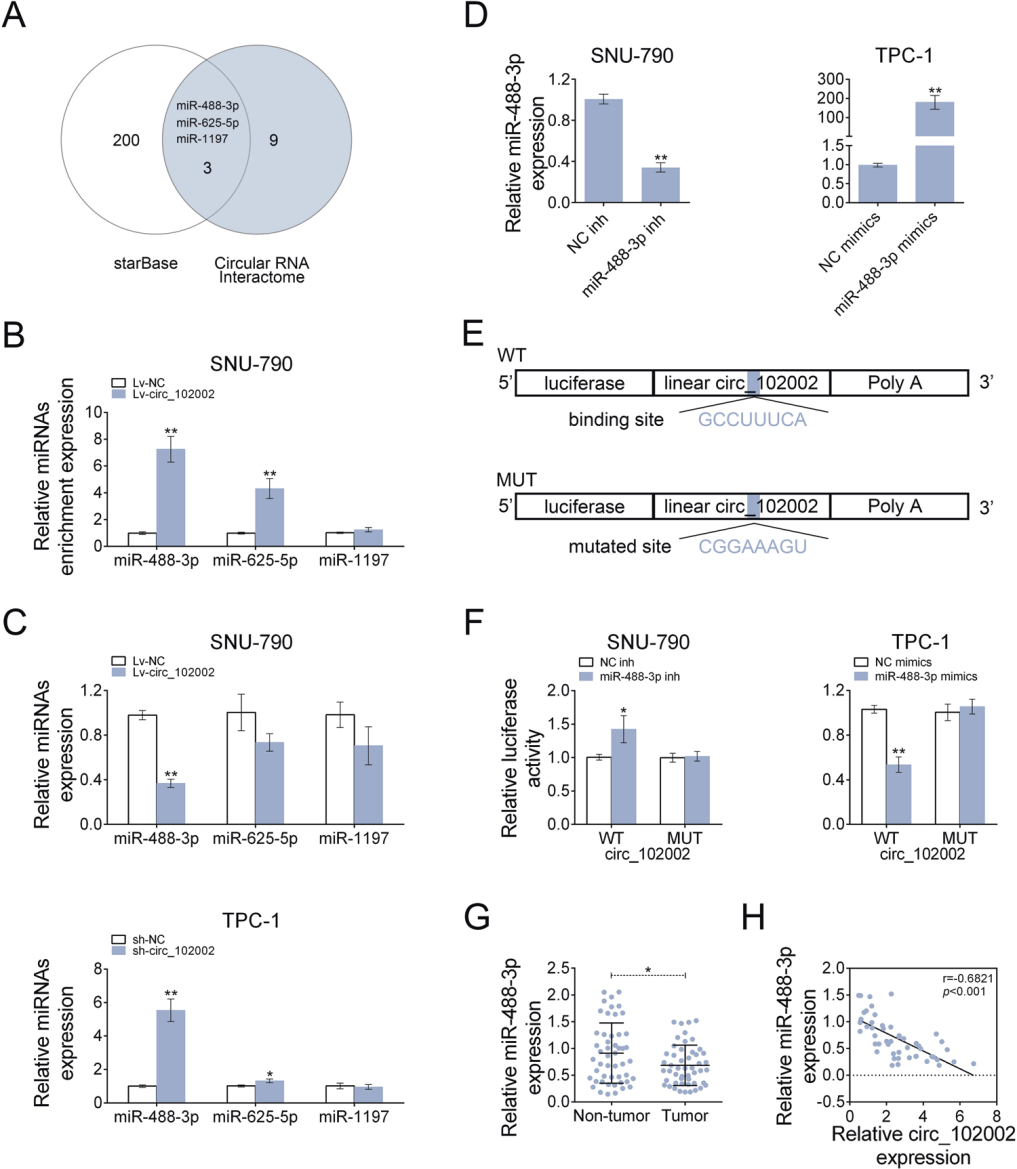
Hsa_circ_102002 could sponge and negatively regulated miR-488-3p. **a** The intersection of potential miRNAs that could be sponged by hsa_circ_102002, as predicted by Circular RNA Interactome and starbase. **b** Relative enrichment levels of miR-488-3p, miR-625-5p and miR-1197 in Lv-NC or Lv-circ_102002 SNU-790 cells, as determined using qRT-PCR. **c** Relative expression levels of miR-488-3p, miR-625-5p, and miR-1197 in Lv-NC or Lv-circ_102002 SNU-790 cells and sh-NC or sh-circ_102002 TPC-1 cells, as determined using qRT-PCR. **d** Relative miR-488-3p levels in SNU-790 cells transfected with NC inh or miR-488-3p inh and in TPC-1 cells transfected with NC mimics or miR-488-3p mimics, as determined using qRT-PCR. **e** A schematic about the design of luciferase assay. The predicted seed-recognition site in the corresponding circ_102002 sequence are marked out. The circ_102002 reporter with mutant binding site was used as a negative control. **f** Relative luciferase activity of the circ_102002 reporter plasmid in SNU-790 cells upon miR-488-3p inh or NC inh transfection and TPC-1 cells upon miR-488-3p mimics or NC mimics transfection. The mutant circ_102002 reporter was used as a negative control. **g** Relative miR-488-3p levels in PTC tissues (tumor, *n* = 50) and normal adjacent tissues (non-tumor, *n* = 50), as determined using qRT-PCR. **h** Pearson’s correlation analysis of the relative expressions between miR-488-3p and circ_102002 (mean ± SEM, *p < 0.05, **p < 0.01).

Circ_102002 was demonstrated to promote EMT and cell migration, but the underlying mechanisms remain unclear. By means of starBase (http://starbase.sysu.edu.cn/starbase2/) and circular RNA Interactome (https://circinteractome.nia.nih.gov/), miR-488-3p, miR-625-5p and miR-1197 were predicted as potential miRNA that might be sponged by circ_102002 (Fig. 4a). RIP result showed that miR-488-3p was most enriched by Ago2 antibody in Lv-circ_102002 SNU-790 cells, which belongs to miRNA-mediated RISC protein complex (Fig. 4b). Among the three miRNAs, miR-488-3p was significantly downregulated in Lv-circ_102002 SNU-790 cells, and was markedly upregulated in sh-circ_102002 TPC-1 cells (Fig. 4c). Thus, the target relationship between miR-488-3p and circ_102002 was studied. As shown in Fig. 4d, miR-488-3p expression was reduced in SNU-790 cells transfected with miR-488-3p inhibitor, while increased in TPC-1 cells transfected with miR-488-3p mimics. Dual luciferase assay was performed to analyze the binding between miR-488-3p and circ_102002 were depicted in Fig. 4e. As Fig. 4f showed, miR-488-3p inhibitor increased the luciferase activity of the circ_102002 reporter plasmids in SNU-790 cells, while miR-488-3p reduced the luciferase activity in TPC-1 cells. However, no significant changes were observed in these cells after the binding sites were mutated. qRT-PCR data demonstrated that miR-488-3p level was markedly downregulated in PTC tissues compared to that in non-tumor tissues (Fig. 4g). Moreover, a negative correlation was observed between miR-488-3p and circ_102002 in clinical PTC tissues according to the Pearson correlation analysis (Fig. 4h). These data revealed that circ_102002 sponged miR-488-3p and negatively regulated it in PTC.

### miR-488-3p could target HAS2 by binding to its 3ʹ-UTR

**Fig. 5:**
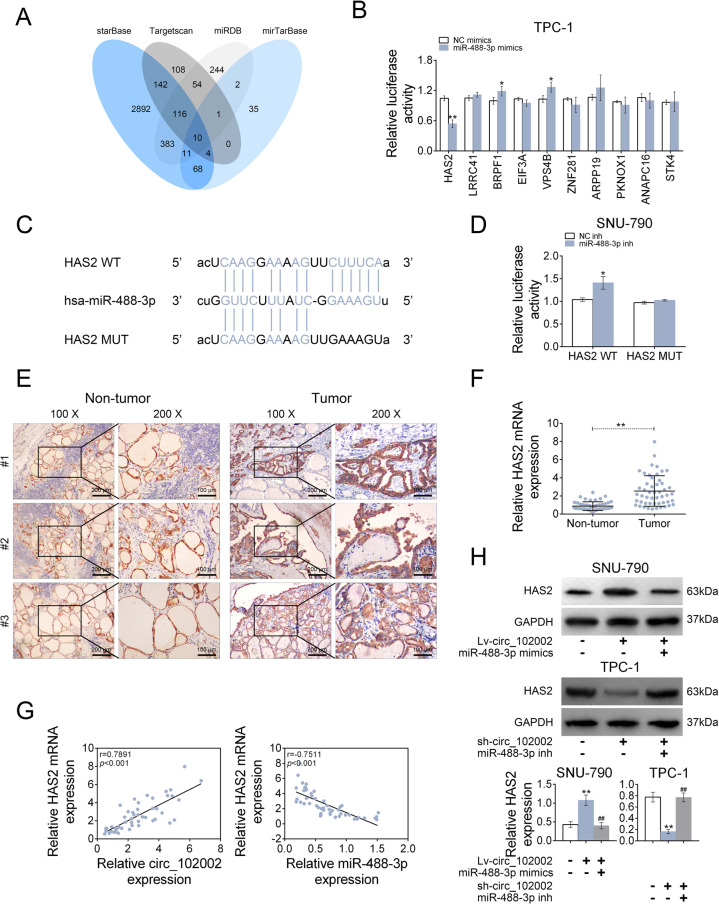
Hsa_circ_102002 negatively regulates miR-488-3p through upregulating HAS2. **a** The intersection of potential genes that could be targeted by miR-488-3p, as predicted by Targetscan, mirTarBase, miRDB and starBase. **b** Relative luciferase activities of the 3ʹ-UTR reporter plasmids (HAS2, LRRC41, BRPF1, EIF3A, VPS4B, ZNF281, ARPP19, PKNOX1, ANAPC16, and STK4) were measured in TPC-1 cells upon transfection of miR-488-3p mimics or NC mimics. **c** Seeds match for miR-488-3p in the 3ʹ-UTR of HAS2. The predicted seed-recognition sites in the HAS2 mRNA sequence and the corresponding miR-488-3p sequence are marked in blue. **d** Relative luciferase activity of the HAS2 3ʹ-UTR reporter plasmid was measured in SNU-790 cells after expressing NC inh or miR-488-3p inh. The mutant HAS2 3ʹ-UTR reporter was used as a negative control. **e** Representative IHC staining of GAPDH and HAS2 in the PTC tissues (tumor, *n* = 50) and normal adjacent tissues. Original magnification, ×40 and ×400. **f** Relative HAS2 mRNA expression levels in PTC tissues (tumor, *n* = 50) and normal adjacent tissues (non-tumor, *n* = 50), as determined using qRT-PCR. **g** Pearson’s correlation analysis of the relative expressions between circ_102002 and HAS2 mRNA, miR-488-3p and HAS2 mRNA in PTC patients. **h** Protein expression levels of HAS2 in Lv-NC + NC mimics, Lv-circ_102002, Lv-circ_102002+miR-488-3p mimics SNU-790 cells, and in sh-NC + NC inh, sh-circ_102002, sh-circ_102002+miR-488-3p inh TPC-1 cells, as determined using western blotting. Bands were quantified and shown in histogram (mean ± SEM, *p < 0.05, **p < 0.01).

To study the target genes of miR-488-3p, starBase, Targetscan, miRDB and mirTarBase were jointly used. The intersection analysis revealed ten potential genes, containing HAS2, LRRC4A, BRPF1, EIF3A, VPS4B, ZNF281, ARPP19, PKNOX1, ANAPC16, and STK4 (Fig. 5a). Among them, the luciferase activity of the HAS2 3ʹ-UTR reporter plasmids was significantly suppressed by miR-488-3p mimics in TPC-1 cells (Fig. 5b). The potential binding sites between miR-488-3p and HAS2 were depicted in Fig. 5c. In addition, miR-488-3p inhibitor significantly increased the luciferase activity of the HAS2 3ʹ-UTR reporter plasmids in SNU-790 cells (Fig. 5d). However, there was no change of luciferase activities after miR-488-3p’s-binding sites were mutated. The IHC (Fig. 5e) and q-PCR (Fig. 5f) results showed that the protein and mRNA expressions of HAS2 were upregulated in clinical PTC tissues. A positive correlation between HAS2 mRNA and circ_102002, and a negative correlation between HAS2 mRNA and miR-488-3p were analyzed in PTC tissues (Fig. 5g) according to Pearson correlation analysis. Furthermore, HAS2 protein expression was increased in Lv-circ_102002 SNU-790 cells, and treatment of miR-488-3p mimics redused the expression of HAS2. Accordingly, HAS2 expression was significantly decreased in sh- circ_102002 TPC-1 cells and its inhibition could be rescued by miR-488-3p inhibitor (Fig. 5h). These findings suggested that miR-488-3p could target HAS2 by binding to its 3ʹ-UTR in PTC.

### Circ_102002’s effects on EMT and migration of PTC cells were mediated by HAS2

**Fig. 6:**
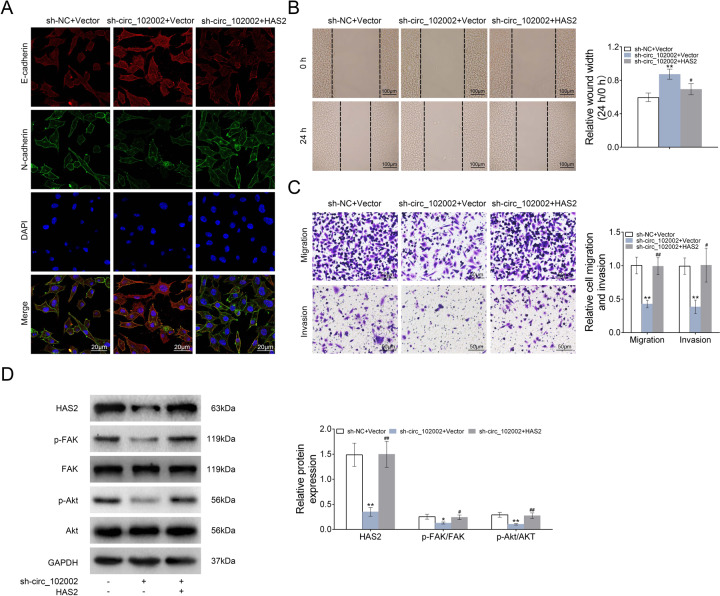
Hsa_circ_102002/HAS2 promoted the epithelial–mesenchymal transformation and migration of PTC cells through regulating FAK/Akt passway. **a** Representative images of E-cadherin and N-cadherin in sh-NC + Vector, sh-circ_102002+Vector, and sh-circ_102002 + HAS2 TPC-1 cells, as determined using IF. (red, E-cadherin; green, N-cadherin; blue, DAPI). **b** Wound scratch healing assay of sh-NC + Vector, sh-circ_102002+Vector, and sh-circ_102002 + HAS2 TPC-1 cells. Quantification of the wound-healing assay was shown as histograms. **c** Representative migration and invasion assay images of sh-NC + Vector, sh-circ_102002+Vector, and sh-circ_102002 + HAS2 TPC-1 cells. The migrated cells were quantified and shown as histograms. **d** Protein expression levels of HAS2, p-FAK, FAK, p-Akt, and Akt in sh-NC + Vector, sh-circ_102002 + Vector, and sh-circ_102002 + HAS2 TPC-1 cells, as determined using western blotting. Bands were quantified and shown in histogram (mean ± SEM, *p < 0.05, **/##*p* < 0.01).

As mentioned above, our results revealed that circ_102002 promote EMT and cell migration in PTC by sponging miR-488-3p. Moreover, miR-488-3p could target HAS2 in PTC. Thus, whether the effects of circ_102002 were mediated by HAS2 was studied. As shown in Fig. 6a, E-cadherin expression was increased and N-cadherin level was downregulated in sh-circ_102002 TPC-1 cells, which could be restored by upregulating HAS2. Results from wound scratch healing assay indicated that the mobility of sh-circ_102002 TPC-1 cells was inhibited and HAS2 overexpression rescued the effect caused by circ_102002 inhibition (Fig. 6b). Transwell assays results showed that the number of migrated and invaded sh-circ_102002 TPC-1 cells was markedly reduced, which was restored by HAS2 overexpression (Fig. 6c). In addition, western blot analysis showed that protein levels of HAS2, p-FAK, and p-Akt were significantly downregulated in sh-circ_102002 TPC-1 cells, while, HAS2 overexpression restore expression levels of p-FAK and p-Akt (Fig. 6d). These findings indicated that circ_102002 affected EMT and migration of PTC cells by regulating HAS2 expression.

### Circ_102002 downregulation inhibited tumor metastasis in vivo

**Fig. 7:**
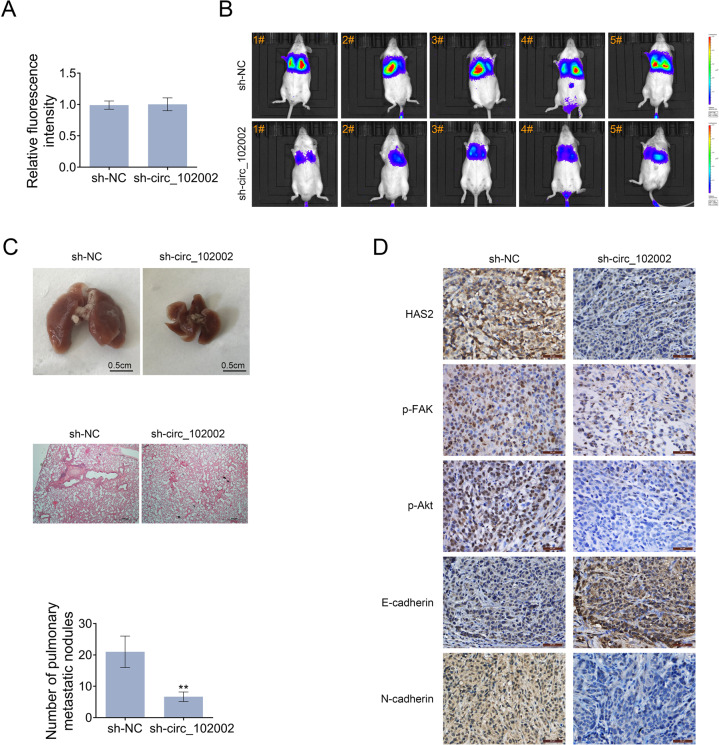
Knockdown of hsa_circ_102002 reduced lung metastasis of PTC in vivo. **a** Relative luciferase activity in sh-NC or sh-circ_102002 TPC-1 cells prior to the cells being injected into mice. **b** Tumor metastasis progression was measured by in vivo luciferase imaging at days 21 after intravenous injection of sh-NC or sh-circ_102002 TPC-1 cells. **c** Representative images and H&E staining analysis of lungs dissected from mice with intravenous injection of sh-NC or sh-circ_102002 TPC-1 cells. Quantification of the lung tumor nodules was shown as histograms. **d** IHC staining of HAS2, p-FAK, p-Akt, E-cadherin, and N-cadherin in the lungs dissected from mice with intravenous injection of sh-NC or sh-circ_102002 TPC-1 cells.

**Fig. 8:**
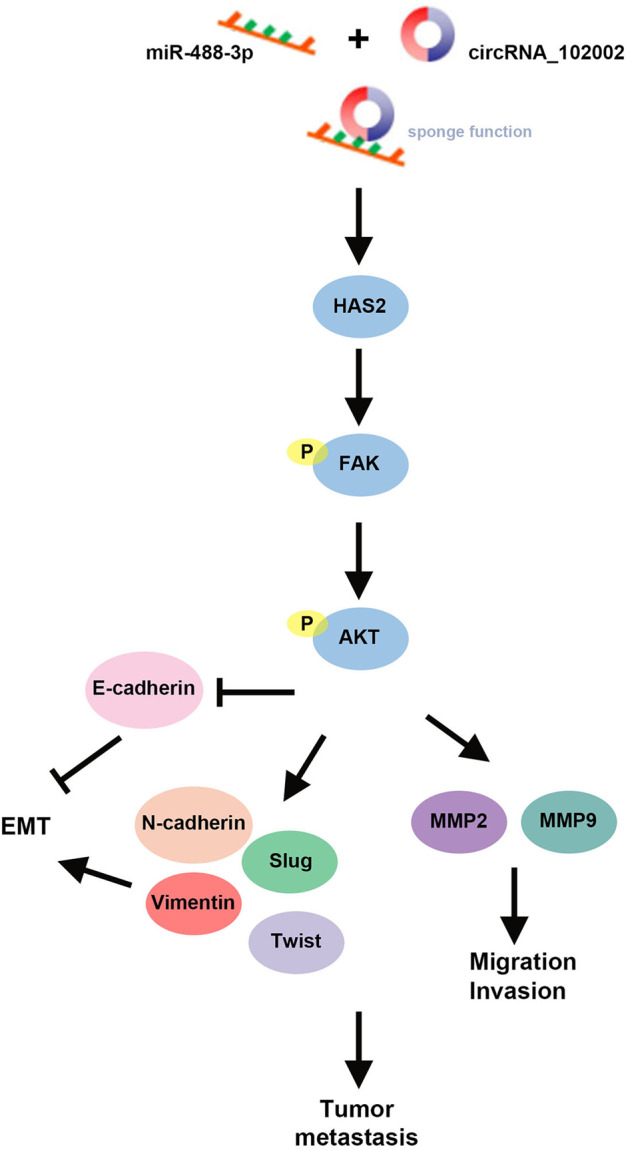
Mechanism diagram. During PTC development, overexpressed has_circ_102002 sponged miR-488-3p, upregulated HAS2 expression and promoted the phosphorylation of FAK and Akt. After that, EMT and cell migration-associated genes were regulated, which ultimately facilitated the tumor metastasis.

On account of that circ_102002 inhibition suppress the EMT and cell migration in vitro, the role of circ_102002 was then studied in a mouse model. To construct metastatic mouse model, sh-NC and sh-circ_102002 TPC-1 cells with a luciferase label were intravenously injected. The luciferase activities in sh-NC and sh-circ_102002 TPC-1 cells exhibited similar prior to the cells being injected into mice (Fig. 7a). Twenty-days days post-implantation, a significant lower degree of lung metastasis were found in mice from sh-circ_102002 group, as determined by an in vivo imaging system (Fig. 7b). The corresponding lungs were excised and histopathological analysis showed that the number of nodules in the lung from sh-circ_102002 group mice was significantly less than that from sh-NC group mice (Fig. 7c). IHC results in Fig. 7d demonstrated that circ_102002 downregulation resulted in decreased expressions of HAS2, p-FAK, and p-Akt in lung tissues of sh-circ_102002 group of mice. Then, upregulated E-cadherin and decreased N-cadherin expression were determined. Altogether, these findings revealed that circ_102002 inhibition could downregulate HAS2 level, suppress the phosphorylation of FAK and Akt, regulate expressions of HAS2, E-cadherin and N-cadherin, and finally inhibit PTC metastasis in vivo (Fig. 8).

## Discussion

We not only demonstrated the simulative effects of circ_102002 on EMT and cell migration, but also identified a regulatory relationship between circ_102002 and miR-488-3p/HAS2. Recently, increasing number of circRNAs are identified and becoming a research hotspot, some of which have been investigated in PTC. For example, circ-ITCH overexpression inhibits PTC cell proliferation and invasion and promotes apoptosis through the miR-22-3p/CBL/β-catenin pathway [27]. In addition, CircZFR promotes the expression of C8orf4 through sponging miR-1261 and contributes to PTC cell proliferation and invasion [28]. These findings suggested that circRNAs usually sponge miRNAs and regulate their target genes. It was predicted that circ_102002 might sponge miR-488-3p, miR-625-5p and miR-1197. Since miR-488-3p was most enriched by circ_102002 probe using RIP assay and downregulated by circ_102002 overexpression. The present study subsequently found that circ_102002 exerts its regulatory functions by downregulating miR-8075. Based on the fact that one circRNA might interact with several molecules at the same time, it is worth further investigation whether there are other miRNAs regulated by circ_102002.

As a newly identified miRNA, miR-488-3p was found as a tumor suppressor in esophageal squamous cell carcinoma and melanoma by respectively targeting ZBTB2 and PRKDC [16, 17]. The features of ZBTB2 have not been reported in PTC. PRKDC encodes DNA-dependent protein kinase catalytic subunit (DNA-PKcs), and its expression was identified positively correlated with the radiosensitivity of PTC [29]. This study showed that miR-488-3p expression was downregulated in PTC and inhibited EMT and cell migration. Among the potential target genes of miR-488-3p, HAS2 stood out because of the significant inhibition efficiency on luciferase reporters caused by miR-488-3p. Therefore, HAS2 is identified as the main target of miR-488-3p in this study. Whether circ_102002/miR-488-3p affects the radiosensitivity of PTC by regulating PRKDC requires further investigations. Furthermore, some noncoding RNAs were found to act as ceRNAs for miR-488-3p. Recently, miR-488-3p has been reported to be sponged by circ_CCDC66 in Hirschsprung’s disease [30], circ_0020123 in non-small cell lung cancer [31], and lncRNA PVT1 in glioma [32] etc. Therefore, it can be speculated that miR-488-3p function as regulatory hub in the progression of these diseases.

As a synthetic enzyme for hyaluronan, HAS2 affects various processes of epithelial-mesenchymal transition and cell migration [20]. HAS2 has been demonstrated to promote tumor progression in some cancers. For instance, HAS2 synthesized hyaluronic acid, enhanced ZEB1 expression and accelerated EMT in breast cancer [19]. HAS2-mediated cancer progression was also indicated in lung cancer [33] and squamous cell carcinoma [34]. Moreover, combined use of HAS2 and HYAL-1 hyaluronidase was able to predict bladder cancer diagnosis and prognosis [35]. So far, the role of HAS2 in PTC has not been reported. In the presented study, the results revealed that overexpression of HAS2 could restore the inhibition of EMT and cell migration caused by circ_102002 downregulation. This process was related to the FAK/PI3K/Akt pathway and was mediated by miRNA-488-3p. Some miRNAs have been reported to target HAS2 in physiopathologic process, which including miR-26b in ovarian granulosa cell apoptosis [36], miR-574 in oocyte maturation [37], and miR-101-3p in synovial sibroblasts [38] etc. However, there was no report about miRNA-regulating the expression of HAS2 during cancer development. Our results for the first time demonstrated miR-488-3p could negatively regulate HAS2 expression and form a molecular signal axis of circ_102002/miR-488-3p/HAS2 in PTC.

The emerging roles for circRNA and miRNAs as tumor suppressors or oncogenes in tumor-related pathways highlight new perspectives for the molecular pathological mechanism, and also offer novel therapeutic tools for cancer treatment [39]. Some miRNAs-based anticancer therapies have entered into clinical testing. For example, MRX34, a miR-34 mimic targeting forkhead box P1 (FOXP1) and BCL2, entered a multicenter phase I trial in 2013 for patients with primary liver cancer and small cell lung cancer. However, this trial was terminated due to immune-related adverse events, which demands new design and efficient delivery [40]. Recently, two phase I clinical trials of the miR-29 mimics targeting histone deacetylase 4 (HDAC4) and MMPs (matrix metalloproteinases) in scleroderma patients and miR-155 inhibitors targeting SHIP (SH2-containing-inositol phosphatase) and RHOA (Ras homolog gene family,member A) in cutaneous T-cell lymphoma patients were initiated and placed great expectations. RNA therapies, when used in combination with other cancer treatments, might have synergistic effects, thereby reducing the dosage and minimizing off-target effects [41, 42]. In this study, the circRNA-miRNA code of circ_102002/miR-488-3p and its effects on epithelial–mesenchymal transition and cell migration were systematically elucidated. Tumor site-specific delivery of modulators of circ_102002/miR-488-3p/HAS2 axis or combination with other approaches will offer new opportunities to design innovative therapeutic strategies for PTC.

In summary, circ_102002 was overexpressed in PTC clinical tissues and cells. EMT and cell migration were promoted by circ_102002 overexpression and inhibited by circ_102002 downregulation. Circ_102002 mainly sponged miR-488-3p and negatively regulated its expression. In addition, HAS2 was identified as a target of miR-488-3p in PTC, which was related to the FAK/PI3K/Akt pathway. Furthermore, the inhibitory effect of downregulation of circ_102002 on PTC growth was evaluated in a mouse xenograft model, which involved miR-488-3p and HAS2 regulation. These findings about the signal axis of circ_102002/miR-488-3p/HAS2 may further elucidate the PTC pathogenesis and improve clinical treatment.

## Supplementary information

Table S1

Table S2

Table S3

## Data Availability

All data generated or analyzed during this study are included in this published article.

## References

[1] Carling T, Udelsman R (2014). Thyroid cancer. Annu Rev Med.

[2] Cabanillas ME, McFadden DG, Durante C (2016). Thyroid cancer. Lancet.

[3] Alzahrani AS, Alkhafaji D, Tuli M, Al-Hindi H, Sadiq BB (2016). Comparison of differentiated thyroid cancer in children and adolescents (</=20 years) with young adults. Clin Endocrinol (Oxf).

[4] Cordioli MI, Moraes L, Cury AN, Cerutti JM (2015). Are we really at the dawn of understanding sporadic pediatric thyroid carcinoma?. Endocr Relat Cancer.

[5] Wang PL, Bao Y, Yee MC, Barrett SP, Hogan GJ, Olsen MN (2014). Circular RNA is expressed across the eukaryotic tree of life. PLoS ONE.

[6] Jeck WR, Sharpless NE (2014). Detecting and characterizing circular RNAs. Nat Biotechnol.

[7] Salzman J, Chen RE, Olsen MN, Wang PL, Brown PO (2013). Cell-type specific features of circular RNA expression. PLoS Genet.

[8] Qu S, Yang X, Li X, Wang J, Gao Y, Shang R (2015). Circular RNA: A new star of noncoding RNAs. Cancer Lett.

[9] Hansen TB, Jensen TI, Clausen BH, Bramsen JB, Finsen B, Damgaard CK (2013). Natural RNA circles function as efficient microRNA sponges. Nature.

[10] Wang H, Yan X, Zhang H, Zhan X (2019). CircRNA circ_0067934 overexpression correlates with poor prognosis and promotes thyroid carcinoma progression. Med Sci Monit.

[11] Bi W, Huang J, Nie C, Liu B, He G, Han J (2018). CircRNA circRNA_102171 promotes papillary thyroid cancer progression through modulating CTNNBIP1-dependent activation of beta-catenin pathway. J Exp Clin Cancer Res.

[12] Peng N, Shi L, Zhang Q, Hu Y, Wang N, Ye H (2017). Microarray profiling of circular RNAs in human papillary thyroid carcinoma. PLoS ONE.

[13] Liang J, Zhang XL, Li S, Xie S, Wang WF, Yu RT (2018). Ubiquitin-specific protease 22 promotes the proliferation, migration and invasion of glioma cells. Cancer Biomark.

[14] Jiang S, Song C, Gu X, Wang M, Miao D, Lv J (2018). Ubiquitin-specific peptidase 22 contributes to colorectal cancer stemness and chemoresistance via wnt/beta-catenin pathway. Cell Physiol Biochem.

[15] Zhao HD, Tang HL, Liu NN, Zhao YL, Liu QQ, Zhu XS (2016). Targeting ubiquitin-specific protease 22 suppresses growth and metastasis of anaplastic thyroid carcinoma. Oncotarget.

[16] Yang Y, Li H, He Z, Xie D, Ni J, Lin X (2019). MicroRNA-488-3p inhibits proliferation and induces apoptosis by targeting ZBTB2 in esophageal squamous cell carcinoma. J Cell Biochem.

[17] Li N, Ma Y, Ma L, Guan Y, Ma L, Yang D (2017). MicroRNA-488-3p sensitizes malignant melanoma cells to cisplatin by targeting PRKDC. Cell Biol Int.

[18] Kosaki R, Watanabe K, Yamaguchi Y (1999). Overproduction of hyaluronan by expression of the hyaluronan synthase Has2 enhances anchorage-independent growth and tumorigenicity. Cancer Res.

[19] Preca BT, Bajdak K, Mock K, Lehmann W, Sundararajan V, Bronsert P (2017). A novel ZEB1/HAS2 positive feedback loop promotes EMT in breast cancer. Oncotarget.

[20] Bernert B, Porsch H, Heldin P (2011). Hyaluronan synthase 2 (HAS2) promotes breast cancer cell invasion by suppression of tissue metalloproteinase inhibitor 1 (TIMP-1). J Biol Chem.

[21] Cheng XB, Kohi S, Koga A, Hirata K, Sato N (2016). Hyaluronan stimulates pancreatic cancer cell motility. Oncotarget.

[22] Kim YH, Lee SB, Shim S, Kim A, Park JH, Jang WS (2019). Hyaluronic acid synthase 2 promotes malignant phenotypes of colorectal cancer cells through transforming growth factor beta signaling. Cancer Sci.

[23] Lemoine N, Mayall E, Jones T, Sheer D, McDermid S, Kendall-Taylor P (1989). Characterisation of human thyroid epithelial cells immortalised in vitro by simian virus 40 DNA transfection. Br J Cancer.

[24] Ma G, Tang M, Wu Y, Xu X, Pan F, Xu R (2016). LncRNAs and miRNAs: potential biomarkers and therapeutic targets for prostate cancer. Am J Transl Res.

[25] Hu Y, Liu Q, Zhang M, Yan Y, Yu H, Ge L (2019). MicroRNA-362-3p attenuates motor deficit following spinal cord injury via targeting paired box gene 2. J Integr Neurosci.

[26] Kastenmayer RJ, Moore RM, Bright AL, Torres-Cruz R, Elkins WR (2012). Select agent and toxin regulations: beyond the eighth edition of the Guide for the Care and Use of Laboratory Animals. J Am Assoc Lab Anim Sci.

[27] Wang M, Chen B, Ru Z, Cong L (2018). CircRNA circ-ITCH suppresses papillary thyroid cancer progression through miR-22-3p/CBL/beta-catenin pathway. Biochem Biophys Res Commun.

[28] Shi J, Ma X, Su Y, Song Y, Tian Y, Yuan S (2018). MiR-31 mediates inflammatory signaling to promote re-epithelialization during skin wound healing. J Invest Dermatol.

[29] Ihara M, Ashizawa K, Shichijo K, Kudo T (2019). Expression of the DNA-dependent protein kinase catalytic subunit is associated with the radiosensitivity of human thyroid cancer cell lines. J Radiat Res.

[30] Wen Z, Shen Q, Zhang H, Su Y, Zhu Z, Chen G (2019). Circular RNA CCDC66 targets DCX to regulate cell proliferation and migration by sponging miR-488-3p in Hirschsprung’s disease. J Cell Physiol.

[31] Wan J, Hao L, Zheng X, Li Z (2019). Circular RNA circ_0020123 promotes non-small cell lung cancer progression by acting as a ceRNA for miR-488-3p to regulate ADAM9 expression. Biochem Biophys Res Commun.

[32] Xue W, Chen J, Liu X, Gong W, Zheng J, Guo X (2018). PVT1 regulates the malignant behaviors of human glioma cells by targeting miR-190a-5p and miR-488-3p. Biochim Biophys Acta Mol Basis Dis.

[33] Song JM, Im J, Nho RS, Han YH, Upadhyaya P, Kassie F (2019). Hyaluronan-CD44/RHAMM interaction-dependent cell proliferation and survival in lung cancer cells. Mol Carcinog.

[34] Wang SJ, Earle C, Wong G, Bourguignon LY (2013). Role of hyaluronan synthase 2 to promote CD44-dependent oral cavity squamous cell carcinoma progression. Head Neck.

[35] Kramer MW, Escudero DO, Lokeshwar SD, Golshani R, Ekwenna OO, Acosta K (2011). Association of hyaluronic acid family members (HAS1, HAS2, and HYAL-1) with bladder cancer diagnosis and prognosis. Cancer.

[36] Liu J, Tu F, Yao W, Li X, Xie Z, Liu H (2016). Conserved miR-26b enhances ovarian granulosa cell apoptosis through HAS2-HA-CD44-Caspase-3 pathway by targeting HAS2. Sci Rep.

[37] Pan B, Toms D, Li J (2018). MicroRNA-574 suppresses oocyte maturation via targeting hyaluronan synthase 2 in porcine cumulus cells. Am J Physiol Cell Physiol.

[38] Feng C, Ji P, Luo P, Xu J (2019). Estrogen-mediated microRNA-101-3p expression represses hyaluronan synthase 2 in synovial fibroblasts from idiopathic condylar resorption patients. J Oral Maxillofac Surg.

[39] Verduci L, Strano S, Yarden Y, Blandino G (2019). The circRNA-microRNA code: emerging implications for cancer diagnosis and treatment. Mol Oncol.

[40] Beg MS, Brenner AJ, Sachdev J, Borad M, Kang YK, Stoudemire J (2017). Phase I study of MRX34, a liposomal miR-34a mimic, administered twice weekly in patients with advanced solid tumors. Invest N. Drugs.

[41] To KKW, Fong W, Tong CWS, Wu M, Yan W, Cho WCS (2020). Advances in the discovery of microRNA-based anticancer therapeutics: latest tools and developments. Expert Opin Drug Discov.

[42] Rupaimoole R, Slack FJ (2017). MicroRNA therapeutics: towards a new era for the management of cancer and other diseases. Nat Rev Drug Discov.

