# Supplementary material for: Hsa_circRNA_102002 facilitates metastasis of papillary thyroid cancer through regulating miR-488-3p/HAS2 axis
Source: Cancer Gene Ther. 2020 Aug 29;28(3):279–93. doi: 10.1038/s41417-020-00218-z (PMC8057948; doi:10.1038/s41417-020-00218-z)
Supplement: Supplementary file 1 — Table S1 [file 41417_2020_218_MOESM1_ESM.doc]

**Table S1** The primers for qRT-PCR assay.

| **Primer Name** | **Primer Sequence (5’-3’)** |
| --- | --- |
| GAPDH RT primer | random primer |
| GAPDH Reverse primer | AAATGAGCCCCAGCCTTC |
| GAPDH Forward primer | AATCCCATCACCATCTTCCAG |
| circ_102002 RT primer | random primer |
| circ_102002 Reverse primer | AGCTGCTTTGTGGACTCCTG |
| circ_102002 Forward primer | GGCGGAAGATCACCACGT |
| USP22 RT primer | random primer |
| USP22 Reverse primer | CGGATAAAGCTGGTGTAGTGG |
| USP22 Forward primer | GAGAGCAGGATGAATGGACAG |
| HAS2 RT primer | random primer |
| HAS2 Reverse primer | AAGAGCTGGATTACTGTGGC |
| HAS2 Forward primer | TCCGAGAATGGCTGTACAATG |
| U6 RT primer | AACGCTTCACGAATTTGCGT |
| U6 Reverse primer | AACGCTTCACGAATTTGCGT |
| U6 Forward primer | CTCGCTTCGGCAGCACA |
| miR-488-3p RT primer | CTCAACTGGTGTCGTGGAGTCGGCAATTCAGTTGAGAGAACCAG |
| miR-488-3p Reverse primer | TGGTGTCGTGGAGTCG |
| miR-488-3p Forward primer | ACACTCCAGCTGGGTTGAAAGGCTATTTT |

**Table S2** Antibodies used in this study.

| **Antibody name** | **Corporation name** | **Catalog** | **Source** | **Poly/monoclonal** | **Dilution ratio (WB)** | **Dilution ratio**  **(IF/IHC)** | **Concentrations** |
| --- | --- | --- | --- | --- | --- | --- | --- |
| Vimentin | Cell Signaling Technology | 5741 | rabbit | monoclonal | 1:1000 | / | 200 μg/mL |
| Slug | Cell Signaling Technology | 9585 | rabbit | monoclonal | 1:1000 | / | 200 μg/mL |
| Twist | Cell Signaling Technology | 69366 | rabbit | monoclonal | 1:1000 | / | 200 μg/mL |
| MMP2 | Cell Signaling Technology | 40994 | rabbit | monoclonal | 1:1000 | / | 200 μg/mL |
| MMP9 | Cell Signaling Technology | 13667 | rabbit | monoclonal | 1:1000 | / | 200 μg/mL |
| HAS2 | Thermo Fisher Scientific | MA5-17087 | mouse | monoclonal | 1:2000 | / | 1 mg/mL |
| p-FAK | Cell Signaling Technology | 8556 | rabbit | monoclonal | 1:1000 | / | 200 μg/mL |
| FAK | Cell Signaling Technology | 71433 | rabbit | monoclonal | 1:1000 | / | 200 μg/mL |
| p-AKT | Cell Signaling Technology | 4060 | rabbit | monoclonal | 1:1000 | / | 200 μg/mL |
| AKT | Cell Signaling Technology | 4691 | rabbit | monoclonal | 1:1000 | / | 200 μg/mL |
| E-cadherin | Cell Signaling Technology | 14472 | mouse | monoclonal | 1:1000 | 1:100 |  |
| N-cadherin | Cell Signaling Technology | 13116 | rabbit | monoclonal | 1:1000 | 1:100 |  |
| GAPDH | Thermo Fisher Scientific | AM4300 | mouse | monoclonal | 1:2000 | 1:100 | 1 mg/mL |
| Goat anti-rabbit IgG | Thermo Fisher Scientific | 31466 | goat | monoclonal | 1:2000 | / | 1 mg/mL |
| Goat anti-mouse IgG | Thermo Fisher Scientific | 31431 | goat | monoclonal | 1:2000 | / | 1 mg/mL |

**Table S3** The primers for plasmid constructs.

| **Primer Name** | **Primer Sequence (5’-3’)** |
| --- | --- |
| circ_102002-p-MIR Reverse primer | GAAGCATGAATTCAAGGTACCCTGGAGGCCATGAAAGGGG |
| circ_102002-p-MIR Forward primer | TAATAACTAAGATCTGGTACCATTCACCAGACCAGAGCACTTGG |
| HAS2-p-MIR Reverse primer | GAAGCATGAATTCAAGGTACCTCTTATCAAAAATATTTTATTTACAAAAAATTAA |
| HAS2-p-MIR Forward primer | TAATAACTAAGATCTGGTACCTCTTCCATGTTTTGACGTTTGC |
