# Supplementary material for: Hsa_circRNA_102002 facilitates metastasis of papillary thyroid cancer through regulating miR-488-3p/HAS2 axis
Source: Cancer Gene Ther. 2020 Aug 29;28(3):279–93. doi: 10.1038/s41417-020-00218-z (PMC8057948; doi:10.1038/s41417-020-00218-z)
Supplement: Supplementary file 2 — Table S2 [file 41417_2020_218_MOESM2_ESM.doc]

**Table S2** Antibodies used in this study.

| **Antibody name** | **Corporation name** | **Catalog** | **Source** | **Poly/monoclonal** | **Dilution ratio (WB)** | **Dilution ratio**  **(IF/IHC)** | **Concentrations** |
| --- | --- | --- | --- | --- | --- | --- | --- |
| Vimentin | Cell Signaling Technology | 5741 | rabbit | monoclonal | 1:1000 | / | 200 μg/mL |
| Slug | Cell Signaling Technology | 9585 | rabbit | monoclonal | 1:1000 | / | 200 μg/mL |
| Twist | Cell Signaling Technology | 69366 | rabbit | monoclonal | 1:1000 | / | 200 μg/mL |
| MMP2 | Cell Signaling Technology | 40994 | rabbit | monoclonal | 1:1000 | / | 200 μg/mL |
| MMP9 | Cell Signaling Technology | 13667 | rabbit | monoclonal | 1:1000 | / | 200 μg/mL |
| HAS2 | Thermo Fisher Scientific | MA5-17087 | mouse | monoclonal | 1:2000 | / | 1 mg/mL |
| p-FAK | Cell Signaling Technology | 8556 | rabbit | monoclonal | 1:1000 | / | 200 μg/mL |
| FAK | Cell Signaling Technology | 71433 | rabbit | monoclonal | 1:1000 | / | 200 μg/mL |
| p-AKT | Cell Signaling Technology | 4060 | rabbit | monoclonal | 1:1000 | / | 200 μg/mL |
| AKT | Cell Signaling Technology | 4691 | rabbit | monoclonal | 1:1000 | / | 200 μg/mL |
| E-cadherin | Cell Signaling Technology | 14472 | mouse | monoclonal | 1:1000 | 1:100 |  |
| N-cadherin | Cell Signaling Technology | 13116 | rabbit | monoclonal | 1:1000 | 1:100 |  |
| GAPDH | Thermo Fisher Scientific | AM4300 | mouse | monoclonal | 1:2000 | 1:100 | 1 mg/mL |
| Goat anti-rabbit IgG | Thermo Fisher Scientific | 31466 | goat | monoclonal | 1:2000 | / | 1 mg/mL |
| Goat anti-mouse IgG | Thermo Fisher Scientific | 31431 | goat | monoclonal | 1:2000 | / | 1 mg/mL |
