# Supplementary material for: Hsa_circRNA_102002 facilitates metastasis of papillary thyroid cancer through regulating miR-488-3p/HAS2 axis
Source: Cancer Gene Ther. 2020 Aug 29;28(3):279–93. doi: 10.1038/s41417-020-00218-z (PMC8057948; doi:10.1038/s41417-020-00218-z)
Supplement: Supplementary file 3 — Table S3 [file 41417_2020_218_MOESM3_ESM.doc]

**Table S3** The primers for plasmid constructs.

| **Primer Name** | **Primer Sequence (5’-3’)** |
| --- | --- |
| circ_102002-p-MIR Reverse primer | GAAGCATGAATTCAAGGTACCCTGGAGGCCATGAAAGGGG |
| circ_102002-p-MIR Forward primer | TAATAACTAAGATCTGGTACCATTCACCAGACCAGAGCACTTGG |
| HAS2-p-MIR Reverse primer | GAAGCATGAATTCAAGGTACCTCTTATCAAAAATATTTTATTTACAAAAAATTAA |
| HAS2-p-MIR Forward primer | TAATAACTAAGATCTGGTACCTCTTCCATGTTTTGACGTTTGC |
